# A comprehensive dataset of the geophilid centipedes of the south-eastern Alps (Chilopoda, Geophilomorpha, Geophilidae s.l.)

**DOI:** 10.3897/BDJ.12.e122144

**Published:** 2024-05-21

**Authors:** Luca Gregnanin, Lucio Bonato

**Affiliations:** 1 Università di Padova, Padova, Italy Università di Padova Padova Italy; 2 National Biodiversity Future Centre, Palermo, Italy National Biodiversity Future Centre Palermo Italy

**Keywords:** Geophilidae, Chilopoda, south-eastern Alps, georeferenced dataset, records, Darwin Core

## Abstract

**Background:**

Centipedes of the family Geophilidae s.l. are widespread in the Holarctic, with the south-eastern part of the European Alps standing out as one of the most investigated regions. However, retrieving the published records for this taxon, even for this region, is challenging, since most of them are sparse in the specialised literature and interpreting them is hampered by the many taxonomic and nomenclatorial changes occurred in the past and recent times.

**New information:**

We assembled and released a dataset of occurrence records of the geophilid species in the south-eastern Alps, including all the published records and many other records present in unpublished catalogues of scientific collections. For each record, we integrated information from all the sources about: locality, date of collection, the taxonomic identifications, number and sex of individuals and available sequences of molecular markers. For all the records, we estimated geographic coordinates of the locality, when not originally provided, based on the information available. We also estimated the accuracy of the position.

The dataset includes 3293 records referred to 39 species, obtained since the first half of the 19^th^ century and up to 2022; 52% of these records have been released publicly for the first time in the dataset here described.

## Introduction

The Geophilidae s.l. is a large lineage of centipedes mainly found throughout the Holarctic with nearly 700 recognised species, some of them traditionally separated in different families (Bonato et al. 2011a, Bonato et al. 2014).

The diversity of geophilids has been studied to varying degrees in different regions. The south-eastern part of the European Alps stands out as one of the most intensely investigated regions. Here, the earliest published records of geophilids date back to the 19^th^ century (e.g., Koch (1847), Koch (1863), Meinert (1870), Fanzago (1874), Fanzago (1876), Fedrizzi (1876a), Fedrizzi (1876b)) and further sampling activities have been carried out until the present. As a result, about 25 species of geophilids are usually reported to occur in the south-eastern Alps (e.g., Zapparoli (1989)), amongst the approximately 130 species known from the whole of Europe (Bonato and Minelli 2014).

However, even within this region, the taxonomy of some groups of species is still imprecise and most probably inaccurate and the actual number of species remains to be clarified (e.g., in the genera *Geophilus* and *Stenotaenia*; Bonato and Minelli (2009), Del Latte et al. (2015)). Moreover, the known distribution of some species across the south-eastern Alps relies only on a few dozen published records and is, therefore, possibly underestimated (e.g., the rarely recorded *Eurygeophiluspinguis*, Bonato et al. (2006)). On the other hand, the occurrence of other species needs confirmation, since it is based on only 1-2 records with uncertain identification (e.g., for *Heniabicarinata*, usually associated with coastal habitats, but recorded in a few cases in mountain regions where other similar species might occur; Magnolini and Bonato (2023)).

Most of the published information about the presence, distribution and ecology of geophilids in the south-eastern Alps is scattered throughout many national or regional journals, in many cases difficult to retrieve, because they are not indexed in modern digital bibliographic catalogues and are not yet available in public digital archives. Indeed, in the last decades, a few synoptic works, with broader taxonomic and geographic scopes, summarised the published records of geophilids species in the south-eastern Alps, providing textual lists of new occurrence records (Zapparoli 1989, Minelli 1992) or a digital catalogue of records mapped on a coarse grid (Zapparoli and Minelli 2005). Since the release of these synopses, however, several taxonomic and nomenclatural changes affected many genera and species (Bonato et al. 2006, Bonato and Minelli 2008, Bonato et al. 2011b, Bonato and Minelli 2014, Peretti and Bonato 2016, Peretti et al. 2022, Bonato et al. 2023).

Here, we present a comprehensive, updated and newly-georeferenced dataset of occurrence records of Geophilidae s.l. from the south-eastern Alps. It includes all the published records, to the best of our knowledge, and many other records retrieved from the catalogues of many major scientific collections hosting relevant specimens (either unpublished catalogues or catalogues available online). For each record, we provided information on locality, date, collector/s, number of individuals recorded, their sex, habitat in which the animals were found and identifiers of published genetic sequences. On top of the original identification, we provided also the different identifications published in subsequent sources and the valid scientific name (according to the taxonomy currently in use) for the species to which each record was assigned in its latest citation.

## Project description

### Title

A comprehensive dataset of the geophilid centipedes of the south-eastern Alps (Chilopoda, Geophilomorpha, Geophilidae s.l.)

### Personnel

Luca Gregnanin, Lucio Bonato.

### Study area description

**Figure 1.**
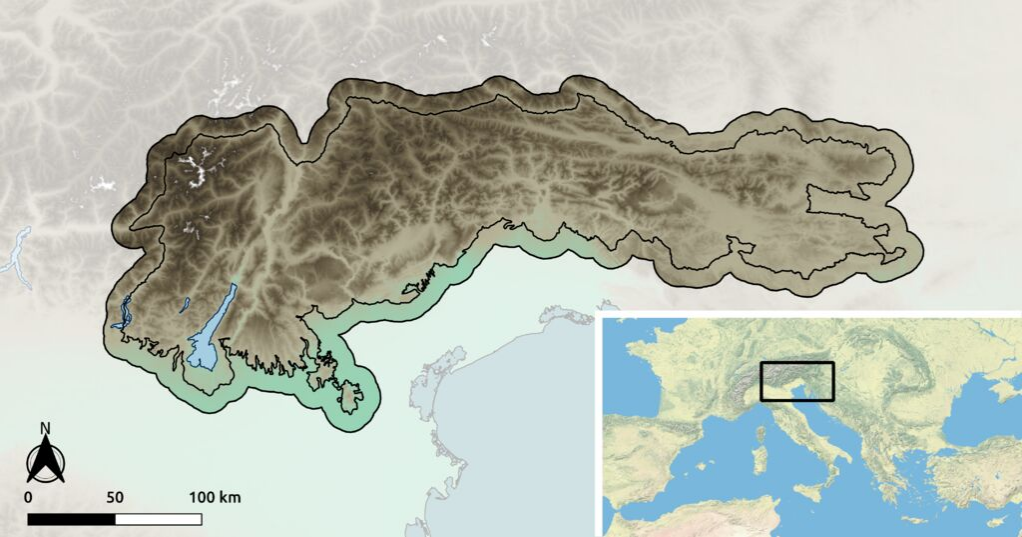
Study area. Inner boundaries follow the SOIUSA sections included in the study area and the southern orographic margins; outer boundaries include a 10 km buffer. Map tiles by ESRI, World Physical Map.

The study area (Fig. 1) covers the south-eastern part of the European Alps. In detail, the study area includes the following "Sections" of the SOIUSA partition of the Alps (Marazzi 2005): Brescia and Garda Prealps, Southern Rhaetian Alps, Venetian Prealps, Dolomites, Carnic and Gailtal Alps, Julian Alps and Prealps, Slovene Prealps, Carinthian–Slovenian Alps. It also encompasses all the major marginal or isolated reliefs along the southern border. To account for the positional uncertainty of the records, we also included in the dataset the records whose estimated position falls within a 10 km marginal buffer (Fig. 1).

## Geographic coverage

### Description

South-eastern part of the European Alps. For further details, see "Study area description".

### Coordinates

45.1287 and 46.9219 Latitude; 15.8613 and 9.9074 Longitude.

## Taxonomic coverage

### Description

Geophilidae Leach, 1816, sensu Bonato et al. (2014), i.e., including the species formerly separated in the families Dignathodontidae Cook, 1896 (within the study area: *Dignathodonmicrocephalus* and *Henia* spp.) and Linotaeniidae Cook, 1899 (within the study area: *Strigamia* spp.).

### Taxa included

**Table:** 

Rank	Scientific Name	
superclass	Myriapoda	
class	Chilopoda	
order	Geophilomorpha	
family	Geophilidae Leach, 1816	
genus	*Acanthogeophilus* Minelli, 1982	
genus	*Clinopodes* C.L. Koch, 1847	
genus	*Dignathodon* Meinert, 1870	
genus	*Eurygeophilus* Verhoeff, 1899	
genus	*Geophilus* Leach, 1814	
genus	*Henia* C.L. Koch, 1847	
genus	*Pachymerium* C.L. Koch, 1847	
genus	*Pleurogeophilus* Verhoeff, 1901	
genus	*Stenotaenia* C.L. Koch, 1847	
genus	*Strigamia* Gray, 1843	
species	*Clinopodescarinthiacus* (Latzel, 1880)	
species	*Clinopodesflavidus* C.L. Koch, 1847	
species	*Clinopodesrodnaensis* (Verhoeff, 1938)	
species	*Clinopodesstrasseri* (Verhoeff, 1938)	
species	*Clinopodesvesubiensis* Bonato, Iorio & Minelli, 2011	
species	*Dignathodonmicrocephalus* (Lucas, 1846)	
species	*Eurygeophiluspinguis* (Brölemann, 1898)	
species	*Geophiluscarnicus* Verhoeff, 1928	
species	*Geophiluscarpophagus* Leach, 1815	
species	*Geophiluselectricus* (Linnaeus,1758)	
species	*Geophilusflavus* (De Geer, 1778)	
species	*Geophilusimpressus* C.L. Koch, 1847	
species	*Geophiluslabrofissus* Verhoeff, 1938	
species	*Geophilusminimus* Verhoeff, 1928	
species	*Geophilusoligopus* (Attems, 1895)	
species	*Geophiluspiae* Minelli, 1983	
species	*Geophilusproximus* C.L. Koch, 1847	
species	*Geophiluspusillifrater* Verhoeff, 1898	
species	*Geophiluspygmaeus* Latzel, 1880	
species	*Geophilustruncorum* Bergsøe & Meinert, 1866	
species	*Heniabicarinata* (Meinert, 1870)	
species	*Heniabrevis* (Silvestri, 1896)	
species	*Heniaillyrica* (Meinert, 1870)	
species	*Heniamontana* (Meinert, 1870)	
species	*Heniavesuviana* (Newport, 1845)	
species	*Pachymeriumferrugineum* (C.L. Koch, 1835)	
kingdom	*Pleurogeophilusmediterraneus* (Meinert, 1870)	
species	*Stenotaenialinearis* (C.L. Koch, 1835)	
species	*Stenotaeniaromana* (Silvestri, 1895)	
species	*Stenotaeniasorrentina* (Attems, 1903)	
species	*Strigamiaacuminata* (Leach, 1816)	
species	*Strigamiacarniolensis* (Verhoeff, 1895)	
species	*Strigamiacrassipes* (C.L. Koch, 1835)	
species	*Strigamiaengadina* (Verhoeff, 1935)	

## Temporal coverage

**Living time period:** Between XIX century and 2022.

## Usage licence

### Usage licence

Other

### IP rights notes

This work is licensed under a Creative Commons Attribution (CC-BY 4.0) Licence.

## Data resources

### Data package title

Geophilidae of south-eastern Alps

### Resource link

https://www.gbif.org/dataset/51b027b6-b6bb-4cac-bb5e-286d3a5a53ee

### Alternative identifiers

https://doi.org/10.15468/zczdmv

### Number of data sets

1

### Data set 1.

#### Data set name

geophilidae_of_south_eastern_alps

#### Data format

TSV (tab-separated) text file

#### Character set

UTF-8

**Data set 1.** 

Column label	Column description
occurrenceID	An identifier for the dwc:Occurrence (as opposed to a particular digital record of the dwc:Occurrence). Value: a text in the format "R####" (#: 0-9).
basisOfRecord	The specific nature of the data record. Value: "MaterialCitation", "PreservedSpecimen".
ownerInstitutionCode	The name in use by the institution (reported as) having ownership of the object(s) or information referred to in the record. Value: a text.
collectionCode	The name identifying the collection from which the record was derived. Value: a text.
catalogNumber	An identifier for the record within the data set or collection. Value: a text.
recordedBy	A list (concatenated and separated) of names of people responsible for recording the original dwc:Occurrence. Value: a list separated by " | ", including the surname and the first letter of the name (when known) of each person; ordered alphabetically.
occurrenceRemarks	Comments or notes about the dwc:Occurrence. Value: a text.
eventDate	The date-time or interval during which a dwc:Event occurred. For occurrences, this is the date-time when the dwc:Event was recorded. Value: a date or time interval conforming ISO 8601-1:2019.
eventRemarks	Comments or notes about the dwc:Event. Value: a possible eventDate for the dwc:Event.
higherGeography	A geographic name less specific than the information captured in the dwc:locality term. Value: the name of the alpine "section" according to the SOIUSA partition of the Alps, preceded by "near" for the records falling outside the conventional borders of the section.
verbatimLocality	The original textual description of the place. Value: a text.
locality	The specific description of the place. Value: the current name of the locality in the main national language(s) of the country to which the locality belongs.
decimalLatitude	The geographic latitude (in decimal degrees, using the spatial reference system WGS84) of the geographic center of a dcterms:Location. Value: a number.
decimalLongitude	The geographic longitude (in decimal degrees, using the spatial reference system WGS84) of the geographic center of a dcterms:Location. Value: a number.
geodeticDatum	The spatial reference system (SRS) upon which the geographic coordinates given in dwc:decimalLatitude and dwc:decimalLongitude are based. Value: for all the records with geographic coordinates, "WGS84".
coordinateUncertaintyInMeteres	The horizontal distance (in meters) from the given dwc:decimalLatitude and dwc:decimalLongitude describing the smallest circle containing the whole of the dcterms:Location. Value: a number.
georeferenceRemarks	Notes or comments about the spatial description determination. Value: a text.
minimumElevationInMeteres	The lower limit of the range of elevation (altitude, usually above sea level), in meters. Value: a number.
maximumElevationInMeteres	The upper limit of the range of elevation (altitude, usually above sea level), in meters. Value: a number.
habitat	A category or description of the habitat in which the dwc:Event occurred. Value: names of plant genera or species, names of phytosociological entities, or other.
verbatimIdentification	A string representing the taxonomic identification as it appeared in the original record. Value: text.
identifiedBy	A list (concatenated and separated) of names of people who assigned the dwc:Taxon to the subject. Value: a list separated by " | ", including the surname and the first letter of the name (when known) of each person; ordered alphabetically; only reported for unpublished records.
dateIdentified	The date on which the subject was determined as representing the dwc:Taxon. Value: a date or time interval conforming ISO 8601-1:2019; only reported for unpublished records.
scientificName	The full scientific name, with authorship and date information. Value: the taxonomic name currently considered valid for the taxon indicated in the verbatimIdentification or for the taxon under which the record was identified in its last citation.
taxonRank	The taxonomic rank of the most specific name in the dwc:scientificName. Value: "family", "genus", "species".
identificationRemarks	Comments or notes about the dwc:Identification. Value: a text.
taxonRemarks	Comments or notes about the taxon or name. Value: a text.
identificationQualifier	A brief phrase or a standard term ("cf.", "aff.") to express the doubts about the dwc:scientificName. Value: "cf.".
individualCount	The number of individuals present at the time of the dwc:Occurrence. Value: a number.
sex	The sex of the biological individual(s) represented in the dwc:Occurrence. Value: "male", "female", their concatenation through " | ".
organismRemarks	Comments or notes about the dwc:Organism instance. Value: possible individualCount for the dwc:Organism. Value: a text.
associatedReferences	A list (concatenated and separated) of identifiers (publication, bibliographic reference, global unique identifier, URI) of literature associated with the dwc:Occurrence. Value: a list separated by " | " of complete citations of all the published sources citing the record; ordered chronologically.
dynamicProperties	A list of additional or amending identifications, dates and localities provided in publications other than the original source. Value: a key:value pair dictionary with keys including author-date references cited in dwc:associatedReferences and values including the additional or amending information.
associatedSequences	A list (concatenated and separated) of identifiers (publication, global unique identifier, URI) of genetic sequence information associated with the dwc:MaterialEntity. Value: a structured text: "marker: [list separated by " | " of GenBank urls].

## Additional information

### Source of records, temporal coverage and contents of the dataset

For our purpose, a record was intended as any report of the finding of one or more individuals of a species in a single location and in a single day.

We searched for all the original records of Geophilidae s.l. in the study area browsing the whole scientific literature reporting records of Chilopoda published up to 2023, however ignoring graduation theses. We also gathered records from the digital catalogues of the major scientific collections of research institutions known to host relevant myriapodological collections and expected to include specimens from the study area: the "Chilobio" centipede collection of the Animal Ecology Group, University of Ljubljana (Ravnjak and Kos 2015); the Bonato-Minelli collection (preserved at the Department of Biology, University of Padova); the collections of the Museo di Storia Naturale di Milano; Museo Civico di Scienze Naturali "E. Caffi" di Bergamo; Museo di Storia Naturale, Verona; Museo Friulano di Storia Naturale, Udine; Naturhistorisches Museum Wien; and Zoologische Staatssammlung München (retrieved from GBIF: Staatliche Naturwissenschaftliche Sammlungen Bayerns (2014)).

The "original source" of a record was intended as the earliest publication reporting the record, if any.

Records were included in the dataset when they were accompanied by at least an indication of the locality (e.g., textual indications, codes of geographical units or geographic coordinates) and a taxonomic identification at the genus level or more precise.

Additional information digitised for each record included any indication, either published in the original source or available from other sources on: time of the recording event (e.g., date, period), habitat (e.g., phytosociological entities, names of plant genera or species type of soil), number and sex of the individuals and the GenBank urls of the available sequences of the main molecular markers employed in molecular taxonomy, phylogeography and population genetics of centipedes, namely the "barcode fragment" of COI, the 16S, 18S and 28S markers, obtained from collected specimens associated with the record. We also queried the BOLD and GenBank databases for additional sequences.

For the name and the structure of the columns of the dataset, we followed the Darwin Core standard (Wieczorek et al. 2012).

### Georeferencing of the records

For each record, the locality where the animals were found was reported as spelled in the original source (in the column "verbatimLocality). A name of the locality was also provided in the main official language(s) of the country to which the locality belongs (in the column "locality"). These latter names were searched in institutional sources (e.g., websites of local administrative institutions) and in topographic maps (e.g., for Italy, the "Carta Topografica d'Italia" map at the scale 1:25000, available as a Web Map Service at http://wms.pcn.minambiente.it/ogc?map=/ms_ogc/ WMS_v1.3/raster/IGM_25000.map).

For each record, the georeferencing of the locality was reported following the "point-radius" method (Wieczorek et al. 2004) by which the locality is associated with: (i) a coordinate pair (latitude and longitude) of the estimated locality and (ii) a linear distance ("radius"), which indicates the uncertainty of the position. The geographic coordinates were reported either as provided in the original or other sources (if available and reliable) or estimated by us by searching in open-access databases (e.g., OpenStreetMap, https://www.openstreetmap.org), or topographic maps. The uncertainty of the coordinates was estimated for all records: when coordinates were provided, but without their uncertainty, this latter was set to 50 m, a reasonable value for a GPS reading uncertainty in mountain settings and for the precision of topographic maps of the region; when coordinates were not provided, their uncertainty was estimated, based on the textual indication of the locality, also taking into account other available information (e.g., the elevation) and the probable meaning given by the authors of the records to the locality names.

### Taxonomy and nomenclature

For each record, we reported the taxonomic name used in the original source (in the column "verbatimIdentification"), other names used for the record in subsequent publications (in the column "dynamicProperties"), and the name currently considered valid for the most recent identification (in the column "scientificName").

For the scientificName, we followed the "Checklist of the Italian Fauna" (Bonato and Minelli 2021) as the main source for taxonomy and nomenclature. For species not included in the Checklist (e.g., species only occurring in the non-Italian part of the study area), we mainly followed Bonato and Minelli (2014). However, we provisionally maintained *Geophiluscarnicus* as a valid species, following Peretti and Bonato (2016). We acknowledged *Geophilusimpressus* as the valid name for the species referred to as *Geophilusalpinus* in the Checklist, following an ICZN opinion (ICZN 2020). We used the name *Clinopodesstrasseri* for a species formerly considered a synonym of *Clinopodescarinthiacus* and recently recognised as a different species (Peretti et al. 2022). Finally, for the species of *Strigamia*, we followed the revised and provisional taxonomy and nomenclature by Bonato et al. (2023), and some of the names (*Strigamiaacuminata*, *Strigamiacarniolensis*, and *Strigamiacrassipes*) are intended as species complexes instead of species.

We reported many records of *Henia* and *Geophilus*, attributed to four putative undescribed species in their original sources, as identified to the genus level in scientificName. They have been flagged with the provisional labels *Henia* sp.1, *Geophilus* sp.1, *Geophilus* sp. 2. and *Geophilus* sp.3 in the column "taxonRemarks".

### Description of the dataset

The dataset includes 3293 records, based on about 7700 collected specimens. They are assigned to 39 species or species–species complexes of Geophilidae s.l., of which four putative species are still undescribed.

A total of 1595 records (48%) were already published, while the remaining 1698 are here released for the first time, being only found in unpublished catalogues of scientific collections. The already published records were found in 86 publications since 1847 (a complete list of references is available in Suppl. material 1).

**Figure 2.**
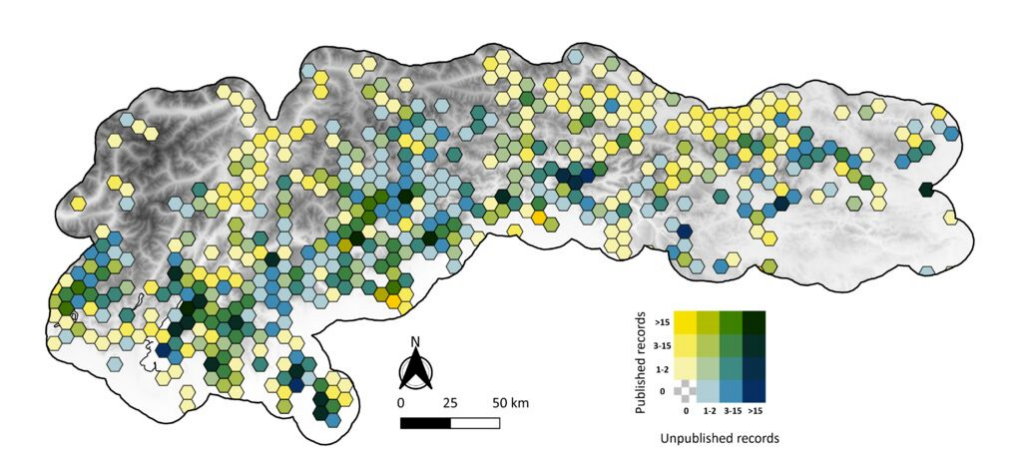
Geographic distribution of the records. Each cell has an area of ~ 40 km^2^. The colour of each cell is associated with the number of records already published (increasing yellow intensity) and to the number of records not yet published (increasing blue intensity). Cells without colour have no records.

The geographic distribution of the records is heterogeneous (Fig. 2): records are denser in the southern part of the study area, a pattern especially evident for the previously unpublished records.

**Figure 3.**
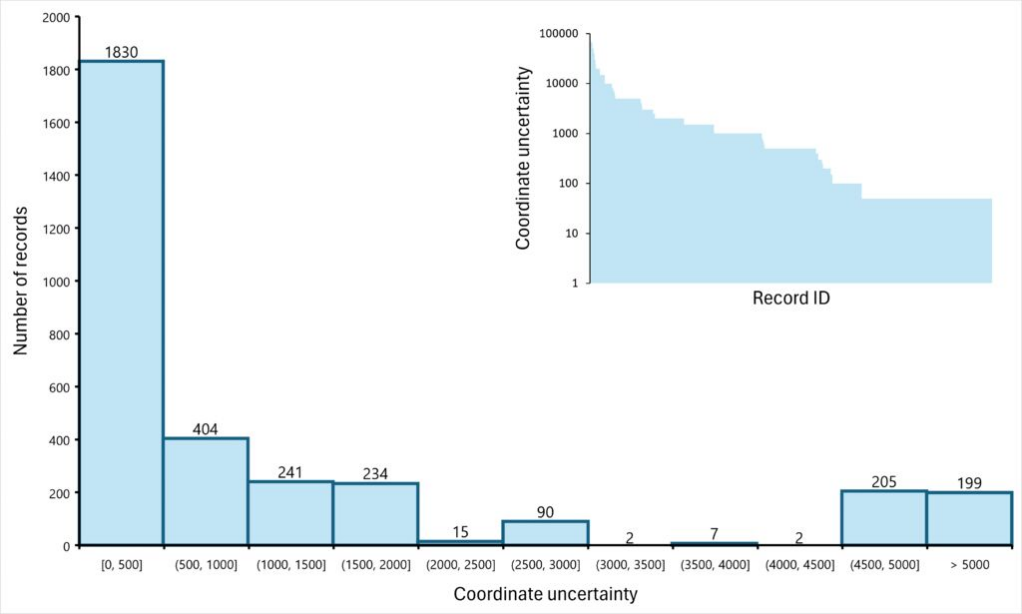
Frequency distribution of the uncertainty of the position of the records (only records with uncertainty < 100 km were georeferenced and are included in this plot). The inset illustrates the uncertainty of the coordinates of each record (records are arranged from the least to the most accurate on the x-axis; note the logarithmic scale on the y-axis).

For 1830 records (56%), the uncertainty of the geographic coordinates was ≤ 500 m (Fig. 3). Instead, 64 records were not georeferenced since the locality indication did not allow us to estimate the coordinates with an uncertainty lower than 100 km.

**Figure 4.**
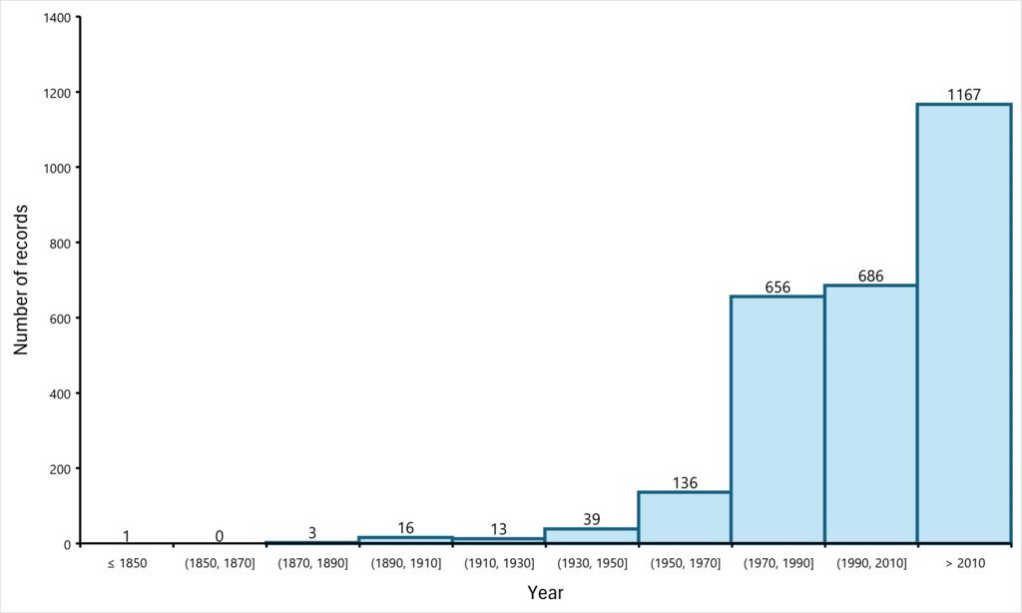
Frequency distribution of the years when the specimens were recorded. Records with uncertain year were excluded when the uncertainty spanned more than two decades.

The oldest record in the dataset dates to 1847 or before (Koch 1847). In the following period, up to about 1970, approximately 500 other records of Geophilidae centipedes from our study area were published, although, in most of the cases, without a precise indication of the recording year. The rate of collection and release of new records increased significantly during the 1970s and since then, more than 2400 records have been collected (Fig. 4).

## Supplementary Material

7CD50F8B-F599-5E51-8DD5-6E22B6771B9A 10.3897/BDJ.12.e122144.suppl1 Supplementary material 1 Bibliographic references of the dataset Data type Bibliographic references Brief description The file includes all the references reporting original records included in the dataset or citing the records with new or amending information. File: oo_1011066.txt https://binary.pensoft.net/file/1011066 Luca Gregnanin, Lucio Bonato
